# In search of innovative potential: The challenge of measuring disruptiveness of research using bibliometric data

**DOI:** 10.1038/s44319-024-00177-8

**Published:** 2024-06-12

**Authors:** Lutz Bornmann, Christoph Ettl, Christian Leibel

**Affiliations:** 1 Science Policy and Strategy Department, Administrative Headquarters of the Max Planck Society, Munich, Germany; 2 Department of Sociology, Ludwig-Maximilians-Universität München, Munich, Germany

**Keywords:** Economics, Law & Politics, History & Philosophy of Science, Science Policy & Publishing

## Abstract

The Disruption index has attracted significant media attention after a study revealed a trend of decreasing disruptiveness in papers and patents. Critiques of the index question these results and highlight issues of inconsistency, time-sensitive biases, and data-induced biases.

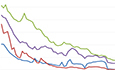

Scientific breakthroughs and disruptive discoveries are the lifeblood of scientific progress. They push the boundaries of our understanding of the world and drive innovation across multiple fields. Innovative discoveries are the driving forces behind the advancement of science and civilization as a whole with far-reaching implications for technology, economy, health, and the environment. Edward Jenner’s development of the smallpox vaccine in 1796 laid the foundation for modern immunization practices. Wilhelm Conrad Röntgen’s accidental discovery of X-rays in 1895 revolutionized medical imaging diagnostics. James Watson’s and Francis Crick’s elucidation of the double helix structure of DNA in 1953 laid the groundwork for genetic engineering, personalized medicine and the Human Genome Project. These disruptive discoveries which occurred accidentally or intentionally were conceptualized by historian and philosopher of science Thomas Kuhn as scientific revolutions: whereas “normal” science operates within the confines of specific paradigms, sudden breakthroughs usher in paradigm shifts, disrupting the existing status quo.

Innovative discoveries are the driving forces behind the advancement of science and civilization as a whole with far-reaching implications for technology, economy, health, and the environment.

The invention of the polymerase chain reaction (PCR) technique in 1983 by Kary Mullis is another example for disruptive science: it enables scientists to replicate and analyse small amounts of genetic material with unprecedented speed and precision, which revolutionized molecular biology and diagnostics. Another discovery with high disruptive potential was the use of the bacterial CRISPR-Cas9 system as a versatile gene editing tool that allows scientists to precisely modify DNA sequences. A further recent example is DeepMind’s AlphaFold AI that demonstrated breakthrough advances in 3D structure prediction of proteins from their amino acid sequences empowering drug discovery, protein engineering, and our understanding of biological processes at the molecular level.

## Looking for disruptive research

These few illustrative examples from the history of science reveal the transformative power of disruptive discoveries and scientific breakthroughs. Since everyone would agree on their importance for science, the question arises how often these discoveries occur and whether they increase or decrease over time. Scientometricians—specialists in quantitative science studies—explore whether discoveries leave traces that can be used to identify and measure their potential for future transformative developments in science.

Scientometricians […] explore whether discoveries leave traces that can be used to identify and measure their potential for future transformative developments in science.

Citation counts are able to measure impact as a proxy for quality but not the disruptive nature of research. Seven years ago, Funk and Owen-Smith (2017) introduced the CD index to measure the extent of technological shifts induced by new patents. Wu et al (2019) transmitted their conceptual idea to published research and proposed to measure the disruptive potential of single papers by using an adapted index (disruption index, DI_1_) to analyse how team size is relevant for publishing disruptive science. Brett and Wang (2020) summarize their results in *Scientific American:* “large teams solve problems; small ones generate new problems to solve” by introducing new ideas and inventions.

Since the introduction of the DI_1_, numerous researchers have employed it to pinpoint the most ground-breaking publications in specific disciplines and fields. In the sciences, DI_1_ has been extensively utilized for numerous fields, including surgery, radiology, breast cancer research, urology, ophthalmology, otolaryngology, trauma research, energy security, information science, and nanoscience.

## Stagnation of science?

**Figure 1:**
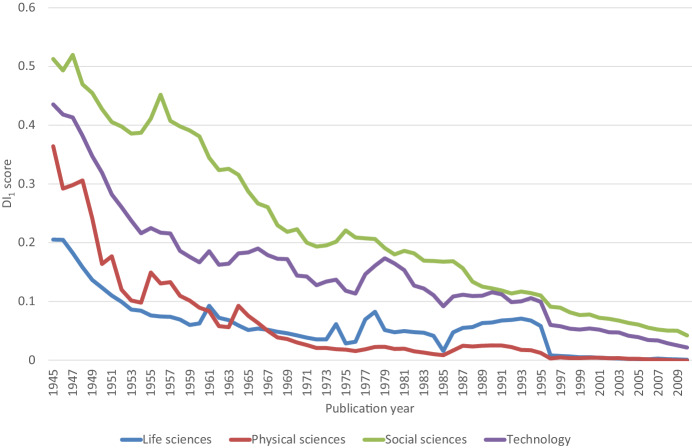
Evolution of average DI_1_ scores based on data published by Park et al (2023). The figure shows a declining trend of disruption scores for papers from all disciplines (life sciences, physical sciences, social sciences, and technology) since the World War II.

But it was another study by Park et al (2023) that stirred the scientific community by asserting that scientific papers and patents have been decreasing in disruptiveness since World War II. Analysing data from 45 million papers and 3.9 million patents, they reported a continuous decline in average disruption scores across all disciplines (Fig. 1). This sparked discussions both within and beyond the scientific community, igniting a public debate on the apparent stagnation of science despite the considerable expansion of the global science system during the past decades.

The results by Park et al (2023) seem to confirm the observations by other scientists (Geman and Geman, 2016; Pauper and Feroz, 2023) that, despite more scientists and more funding, scientific breakthroughs have become rare: “more science, but less world-changing science” (Piper, 2023). Funk and Park (2023) mention the “low-hanging fruit” theory as a possible reason for this decline: easy-to-achieve insights have already been gained in the past and are no longer detectable by recent researchers. Other possible reasons are: the increasing training time (effort) required to reach the frontiers of fields—if the frontiers are achieved at all by young researchers; and possibly increasing tendencies of researchers to favor lower-risk research in the competition for research funding, which encourages conservative decision-making. Kamerlin and Lynn (2023) additionally mentions the “ever-expanding administrative burdens, the paucity of funding and hypercompetition, the lack of jobs which pushes the brightest minds out of academia or research.”

While the notion that both patents and papers are experiencing “less bang per buck” is undoubtedly striking, it is crucial not to draw hasty conclusions, especially for science policy. Park et al (2023) themselves caution that “even though research to date supports the validity of the CD index [referred to as DI_1_ in this comment], it is a relatively new index of innovative activity and will benefit from future work on its behaviour and properties”. Consequently, any meaningful discussion about their findings, or any other study involving the DI_1_, necessitates a comprehensive understanding of the index’s properties and limitations. Numerous empirical studies have delved into these aspects since 2019, contributing to a nuanced interpretation of the spectacular results presented by Park et al (2023). To comprehend these results, it is imperative to first grasp the precise methodology employed in calculating the DI_1_.

While the notion that both patents and papers are experiencing “less bang per buck” is undoubtedly striking, it is crucial not to draw hasty conclusions, especially for science policy.

## Definition of the disruption index

**Figure 2:**
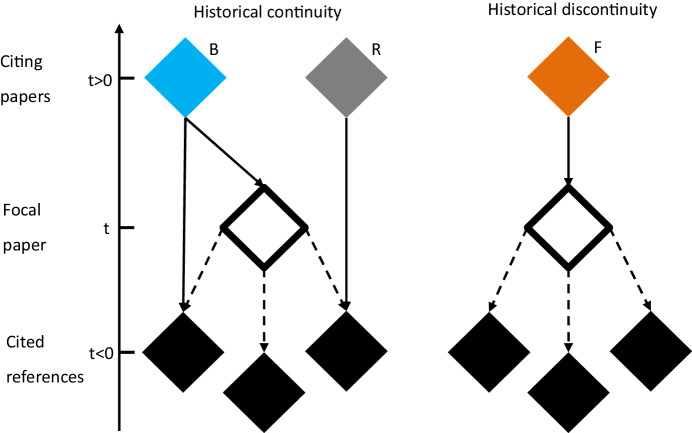
Calculation of the DI_1_ in a tripartite network. The illustration is based on Funk and Owen-Smith (2017) and Leibel and Bornmann (2024).

The DI_1_ is based on a citation network around a focal paper (FP) (Fig. 2). The index stands in the tradition of using betweenness centrality to measure the importance of nodes in networks. In citation networks, betweenness centrality can be measured via bibliographic coupling links that connect publications with citations of the the same publications. If, for example, publication A and publication B both cite publication C, then there is a bibliographic coupling link between A and B. The DI_1_ then differentiates between three types of citing papers (bibliographic coupling links). Citing papers of type R (for “reference”) which have been published after the FP, only cite the FP’s cited references but not the FP itself. Papers of type B (standing for “both”) cite both the FP and at least one of the FP’s cited references. Papers of type F (standing for “focal”) cite only the FP and none of its cited references. The DI_1_ is equivalent to the following formula:

$${{{{DI}}}}_{1}=\frac{{N}_{{{F}}}-{N}_{{{B}}}}{{N}_{{{F}}}+{N}_{{{B}}}+{N}_{{{{R}}}_{t > 0}}}$$

${N}_{{{F}}}$, ${N}_{{{B}}}$, and ${N}_{{{{R}}}_{t > 0}}$ are the total number of papers in sets F, B, and R, respectively (Fig. 2).

The idea is that bibliographic coupling links between an FP and its citing papers generate historical continuity, because ${N}_{{{B}}}$ and ${N}_{{{{R}}}_{t > 0}}$ connect the more recent research to research that predates the FP. Bibliographic coupling links indicate that the FP does not diminish the importance of previous research, which suggests that the FP is consolidating. In turn, the absence of bibliographic coupling links (${N}_{{{F}}}$) represents historical discontinuity. An FP is disruptive if research that predates the FP is no longer relevant for future research.

The DI_1_ operates within a scale ranging from −1 to 1, where negative values would signify consolidating papers, while positive values indicate disruptive papers. It is essential to note that the DI_1_ is a relative index, treating disruption and consolidation as opposing concepts. In fact though from an absolute standpoint, an FP could exhibit both numerous bibliographic coupling links indicative of consolidating science and a large ${N}_{F}$ indicating disruptive science. This would categorize the FP as both highly disruptive and highly consolidating. From the relative standpoint of the DI_1_, however, such an interplay between disruption and consolidation adheres to a zero-sum game: a publication cannot be simultaneously disruptive and consolidating. For instance, an article with a DI_1_ score of 0.5 is expected to be more disruptive and less consolidating than an article with a DI_1_ score of 0. Conversely, an article with a DI_1_ score of 0.3 is less disruptive and more consolidating than an article with a DI_1_ score of 0.4.

## Weaknesses of the disruption index

The DI_1_ assumes a normative citation behavior of authors, namely that they cite papers which have cognitively influenced them. However, if an author decides, consciously or unconsciously, to ignore important preliminary publications in the FP, the paper would probably have a higher positive index score than would be justified by the presented research. Indeed, many studies in scientometrics have demonstrated that citing scientists do not follow a normative citation behavior (Tahamtan and Bornmann, 2018, 2019). When interpreting empirical results based on the DI_1_, it is therefore important to keep in mind that deviations from the normative theory in citing behavior may distort results on the disruptive potential of FPs.

As long as an FP continues to accumulate additional citations, the citation network undergoes changes, leading to significant variations in disruption scores depending on the time scale. Beyond temporal considerations, the DI_1_ may also be influenced by the total number of the FP’s cited references and the citation impact of the FP’s cited references. If an FP contains numerous cited references and these references have garnered substantial citations, it increases the likelihood that citing papers will cite at least one of the FP’s references.

Ruan et al (2021) found that “low coverage of a citation database boosts D values [(DI_1_ scores)]. Specifically, low coverage of non-journal literature in the Web of Science (WoS[, Clarivate]) boosted D values in social sciences, and the exclusion of non-Chinese language literature in the Chinese Social Sciences Citation Index (CSSCI) resulted in the inflation of D values in Chinese language literature.” As such publications—not covered as source items in literature databases—are not factored into disruption score calculations, an FP exclusively citing books with ${N}_{{{{R}}}_{t > 0}}={N}_{{{B}}}=0$ would be assigned the highest disruption score of 1 in the WoS: a data artefact. A publication in the CSSCI solely citing English literature would probably receive a disruption score of 1 due to its exclusion of citations to non-English publications. Expanding on this line of argument, Liang et al (2022) emphasized that the lack of coverage in bibliometric databases such as Scopus (Elsevier) disproportionately affects older publications compared to more recent literature in the index calculation.

## Variants of the disruption index

Since the inception of the DI_1_, researchers have proposed various variants to address these and other weaknesses in the calculation, use, and interpretation of the new index. Among these is a new index type proposed by Bornmann et al (2020) which introduces the idea of a threshold *I*, such that only citing papers that cite at least *I* of the FP’s cited references are considered in the calculation of ${N}_{{{B}}}$. This modification aims to mitigate biases stemming from highly cited papers in a field that may be rhetorically cited. Rhetorically cited papers with high impact can diminish the DI_1_ score of the FP. The authors recommend a threshold of *I* = 5 for the index (resulting in DI_5_) that would exclude certain citing papers: papers that cite fewer than five of the FP’s cited references in ${N}_{{{B}}}$. The exclusion emphasizes those citing papers relying more heavily on the FP’s references. Numerous studies have investigated the validity of the DI_1_, DI_5_, and other variants. Convergent validity can be assessed through two approaches: comparing the results with those of other metrics measuring similar concepts; or assessing how well the metric aligns with expert evaluations of the same or similar concepts. Leibel’s and Bornmann’s (2024) review of the literature on the disruption index in scientometrics concludes that “the literature on convergent validity is not entirely conclusive.”

Current research on the DI_1_ and its variants reveals two key insights. First, the DI_1_ and its variants facilitate the exploration of complex research questions in science of science studies that demand extensive bibliometric data. Notable studies by Wu et al (2019), Park et al (2023), and Lin et al (2023) heavily relied on the DI_1_. Second, it is evident that the DI_1_ and its variants are influenced by a lack of coverage in literature databases related to time, discipline, document type, and language. This raises concerns about the potential distortion of results.

On a positive note, the DI_1_ and its variants exhibit versatility, offering researchers multiple options to address their weaknesses. To enhance reliability (validity), disruption scores are increasingly calculated only for publications with a minimum number of citations and cited references. It is advisable not to compute disruption scores for publications from very early years. Due to the potential unreliability of results with a short citation window, Bornmann and Tekles (2019) suggest a citation window of at least years, aligning with bibliometric standards. Since a three-year period may not guarantee reliable (and valid) results for articles continuing to accrue citations long after publication, researchers should transparently justify their choice of the citation window.

To enhance validity, disruption scores are increasingly calculated only for publications with a minimum number of citations and cited references.

## An example of applying a DI variant

**Figure 3:**
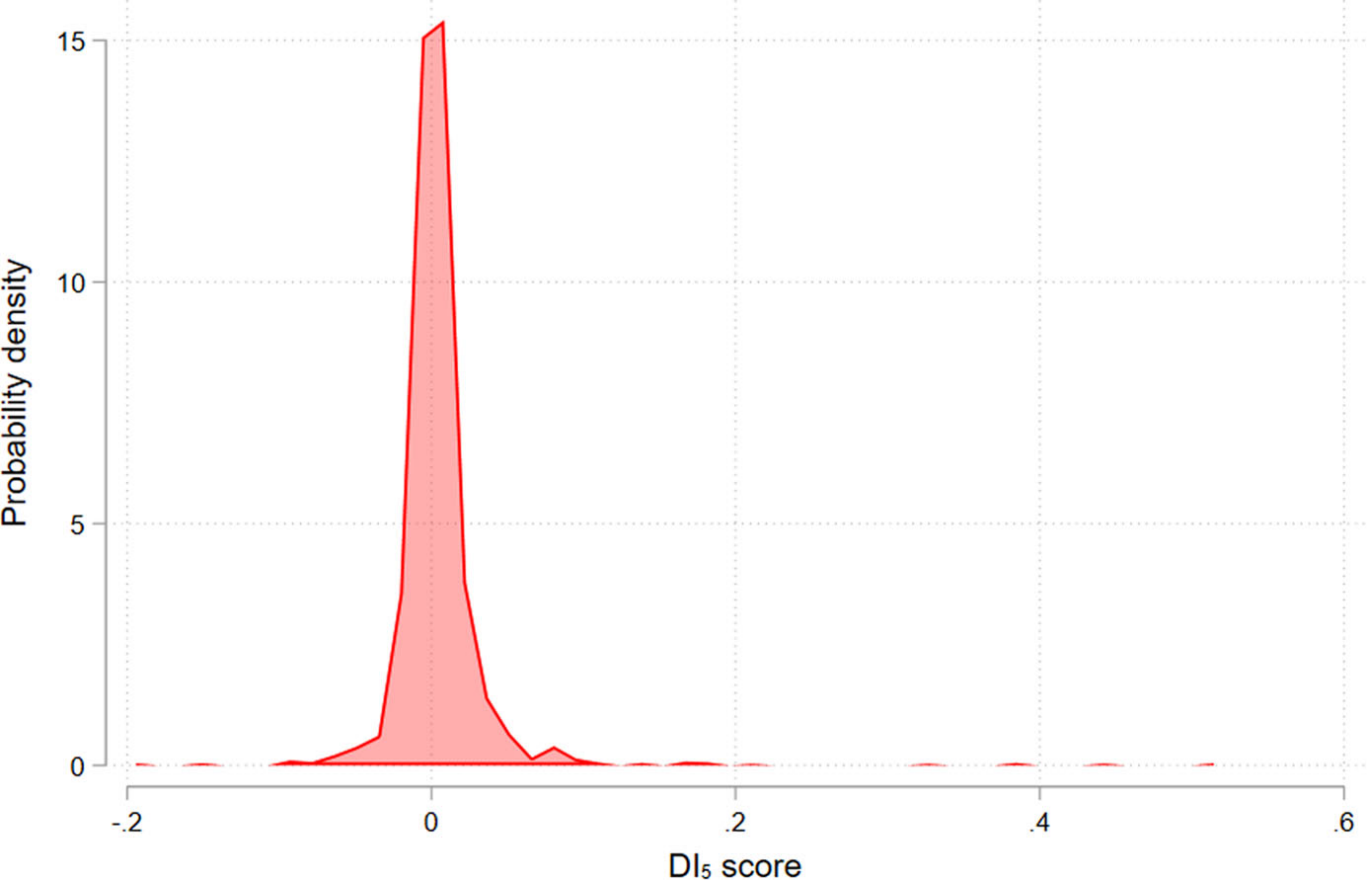
Distribution of DI_5_ scores of papers published in *Nature* and *Science* between 2016 and 2020. The figure shows the distribution of the scores for 8560 papers with at least 10 citations and 10 cited references.

Following suggestions concerning the minimum number of citations and cited references, the minimum citation window, and using the DI_5_ variant, we exemplary calculated index scores for papers published in *Nature* and *Science* between 2016 and 2020. We used data from our in-house database at the Max Planck Society based on the WoS. Figure 3 shows the distribution of the DI_5_ scores for 8560 papers with at least 10 citations and 10 cited references. This set is also restricted to papers for which at least 75% of the cited references are covered as source items in the in-house database (linked references). Note that the in-house database only covers publications back to the year 1980.

Figure 3 shows that the vast majority of papers have DI_5_ scores around 0, which suggests that most of the papers published in *Nature* and *Science* are neither disruptive nor consolidating in terms of the DI_5_. Similar results have been reported for many other datasets. The minimum score in the dataset is −0.2, and the maximum score is 0.5. The most disruptive paper, titled “A bacterium that degrades and assimilates poly(ethylene terephthalate) (PET)”, was published in *Science* by Yoshida et al (2016) and has been cited 1687 times to date (retrieved from Scopus on May 7, 2024). It reports the discovery of a bacterium, *Ideonella sakaiensis*, that completely degrades and assimilates polyethylene terephthalate (PET) as its sole carbon source. The discovery has important implications for PET removal and recycling, and principles of enzyme evolution.

There is no other paper in the dataset with scores greater than 0.5; only 4 papers have scores of at least 0.3. Many papers with high disruption scores either have less than 10 cited references and/or citations or fell below the 75% threshold share of linked cited references. The high DI_5_ scores for many papers in the initial dataset probably point to data artefacts. After their removal, only a low number of (highly) disruptive papers remain in the dataset. These results may question the validity of the DI_5_ (but also of the DI_1_ with very similar results), since one would expect more disruptive papers published in *Nature* and *Science* with scores greater than 0.5.

## Conclusion

Our empirical results indicate that further research is necessary on the reliability and validity of the DI_1_ and its variants. We need to know the exact thresholds (data) and literature databases for calculating reliable scores. It will require studies to know for which publication years and disciplines meaningful scores can be calculated. Since the DI_1_ and its variants have received a lot of attention in science and science policy and have been applied empirically in several disciplines, one should keep in mind that disruption scores are susceptible to manipulation, probably even more so than the established citation impact scores. The indices are “heavily dependent on the references cited in the focal paper. For instance, a researcher could artificially inflate their disruptive citation by citing references with minimal citation or by citing fewer references or even omitting references altogether” (Yang et al, 2023).

... one should keep in mind that disruption scores are susceptible to manipulation, probably even more so than the established citation impact scores.

### Supplementary information

Peer Review File
