# Supplementary material for: In search of innovative potential: The challenge of measuring disruptiveness of research using bibliometric data
Source: EMBO Rep. 2024 Jun 12;25(7):2837–41. doi: 10.1038/s44319-024-00177-8 (PMC11239872; doi:10.1038/s44319-024-00177-8)
Supplement: Supplementary file 1 — Peer Review File [file 44319_2024_177_MOESM1_ESM.pdf]

EMBO reports

In search of innovative potential

Lutz Bornmann, Christoph Ettl, and Christian Leibel

Corresponding author(s): Lutz Bornmann (bornmann@gv.mpg.de)

Review Timeline:

Submission Date:	31st May 24
Accepted:	5th Jun 24

Editor: Holger Breithaupt

Transaction Report: This article was editorially reviewed.
